# Comparative Analysis of the Biomechanical Characteristics After Different Minimally Invasive Surgeries for Cervical Spondylopathy: A Finite Element Analysis

**DOI:** 10.3389/fbioe.2021.772853

**Published:** 2021-12-16

**Authors:** Tao He, Jun Zhang, Tong Yu, Jiuping Wu, Tianyang Yuan, Rui Liu, Zhihe Yun, Haorui Du, Le Qi, Junyan An, Wu Xue, Xinyu Nie, Qinyi Liu

**Affiliations:** Department of Spine Surgery, The Second Hospital of Jilin University, Jilin University, Changchun, China

**Keywords:** anterior transdiscal approach of endoscopic cervical discectomy, posterior endoscopic cervical foraminotomy, microsurgical anterior cervical foraminotomy, anterior transcorporeal approach of endoscopic cervical discectomy, cervical minimally invasive surgery, biomechanics, finite element analysis

## Abstract

Minimally invasive surgeries, including posterior endoscopic cervical foraminotomy (PECF), microsurgical anterior cervical foraminotomy (MACF), anterior transdiscal approach of endoscopic cervical discectomy (ATd-ECD), and anterior transcorporeal approach of endoscopic cervical discectomy (ATc-ECD), have obtained positive results for cervical spondylotic radiculopathy. Nonetheless, there is a lack of comparison among them regarding their biomechanical performance. The purpose of this study is to investigate the biomechanical changes of operated and adjacent segments after minimally invasive surgeries compared to a normal cervical spine. A three-dimensional model of normal cervical vertebrae C3–C7 was established using finite element analysis. Afterwards, four surgical models (PECF, MACF, ATd-ECD, and ATc-ECD) were constructed on the basis of the normal model. Identical load conditions were applied to simulate flexion, extension, lateral bending, and axial rotation of the cervical spine. We calculated the range of motion (ROM), intradiscal pressure (IDP), annulus fibrosus pressure (AFP), uncovertebral joints contact pressure (CPRESS), and facet joints CPRESS under different motions. For all circumstances, ATc-ECD was close to the normal cervical spine model, whereas ATd-ECD significantly increased ROM and joints CPRESS and decreased IDP in the operated segment. PECF increased more the operated segment ROM than did the MACF, but the MACF obtained maximum IDP and AFP. Except for ATc-ECD, the other models increased joints CPRESS of the operated segment. For adjacent segments, ROM, IDP, and joints CPRESS showed a downward trend in all models. All models showed good biomechanical stability. With their combination biomechanics, safety, and conditions of application, PECF and ATc-ECD could be appropriate choices for cervical spondylotic radiculopathy.

## Introduction

Cervical spondylotic radiculopathy is usually characterized by pain and numbness of the neck, shoulders, and arms as well as restriction of cervical movement, which significantly decreases quality of life for patients (Yuchi et al., 2019; Chen et al., 2020). Anterior cervical discectomy and fusion has turned into a standard procedure for cervical spondylotic radiculopathy because of its safety, effectiveness, and high fusion rate since the 1950s (Ruetten et al., 2009; Yang et al., 2014; Ren et al., 2019), notwithstanding that it may cause some problems such as degeneration of adjacent segments, loss of intervertebral disc height, and pseudarthrosis formation (Ruetten et al., 2009; Yang et al., 2014; Yuchi et al., 2019; Chen et al., 2020). With the development of full-endoscopic cervical discectomy, the complications of anterior cervical discectomy and fusion were managed appropriately (Ruetten et al., 2009; Wu et al., 2018; Ahn, 2020). Full-endoscopic cervical discectomy is generally divided into two types, i.e., anterior transdiscal approach of endoscopic cervical discectomy (ATd-ECD) and posterior endoscopic cervical foraminotomy (PECF) (Yang et al., 2014; Ren et al., 2020). PECF is an indirect decompression of the technique through the posterior approach, which requires the removal of the partial bony structure and soft tissue with a radius of 3–4 mm around the V-point (inferior margin of the cephalic lamina, superior margin of the caudal lamina, and the medial border of facet joints [FJ]) to decompress the nerve root (Kim et al., 2015; Ahn, 2016; Won et al., 2017; Wu P. H. et al., 2021). The ATd-ECD technique can achieve precision and direction of approach using contrast agents, but a tunnel needs to be built in the intervertebral disc; after that, the endoscopic instruments remove the protruded disc through the intervertebral space (Lee and Lee, 2014; Quillo-Olvera et al., 2018; Haijun et al., 2021). Nowadays, PECF and ATd-ECD have gradually become alternative options for spine surgeons in treatment of cervical disc herniation because of their good postoperative stability and high clinical success rate (Ruetten et al., 2008; Yang et al., 2014).

ATd-ECD generates greater iatrogenic disc injury and causes intervertebral space decrease (Ren et al., 2020). With regard to better disc preservation, a similar technique named anterior transcorporeal approach of endoscopic cervical discectomy (ATc-ECD) was used subsequently (Choi et al., 2007). ATc-ECD can relieve the compression of the nerve root by drilling a hole in the vertebral body, which avoids the unnecessary destruction of the intervertebral disc and bony stabilizers because it can reach the region of the protruded disc or uncovertebral osteophyte through the bone tunnel (Choi et al., 2007; Kim et al., 2011; Umebayashi et al., 2013; Deng et al., 2016; Wu et al., 2018; Chen et al., 2021). Ren et al. (2020) reported that the rate of excellent or good results reached up to 91.4%, and intervertebral space decrease was reported after ATc-ECD. However, it was not revealed whether intervertebral space decrease after ATc-ECD causes the apparent biomechanical changes of structures, like the intervertebral disc, FJs, and uncovertebral joints (UJs). Previous experiments focused primarily on vertebral strength change after operation (Umebayashi et al., 2013; Ren et al., 2020); thus, the biomechanics of ATc-ECD need to be further explored in detail.

Aside from a herniated disc, secondary osseous foraminal stenosis resulting from UJ osteophytes can also potentially induce cervical spondylotic radiculopathy (Boreadis and Gershon-Cohen, 1956; Snyder et al., 2007; Brismée et al., 2009; Clifton et al., 2020). Previous research demonstrated that the direct decompression of foraminal stenosis by resecting UJ effectively relieved the symptoms of nerve compression (Jho, 1997; Saringer et al., 2002; Saringer et al., 2003; Kotil and Bilge, 2008). MACF is regarded as an alternative functional surgery and can preserve segmental motion compared with anterior cervical discectomy and fusion (Maduri et al., 2020). And it can also represent a supplement surgery to better improve arm pain after conducting anterior cervical discectomy and fusion (Lee et al., 2018; Clifton et al., 2020; Noh et al., 2020). A key step of MACF is the necessary removal of hypertrophied UJs to expand the intervertebral foramen and decompress the nerve root (Taşçioğlu et al., 2001; Clifton et al., 2019). However, UJs are regarded as a stabilizer to limit cervical posterior translation and lateral bending, and resection of UJs will disrupt stability and may add a load on other bearing structures (Kotani et al., 1998; Wang et al., 2016). Kotani et al. (1998) divided UJs into three parts, namely, the posterior foraminal part, the posterior half, and the anterior half, and separately analyzed the stability of each part; however, the detailed biomechanical responses inside of the cervical spine were not exposed.

Previous studies showed that PECF obtained better postoperative stability than did ATd-ECD (Yuchi et al., 2019; Chen et al., 2020). Still, there is a lack of comparative study of biomechanics for the four aforementioned minimally invasive surgeries, and it is unclear which surgery represents optimal stability. Besides, the variations of UJs have not been discussed after cervical surgeries. Due to limitations of technology, it is currently impossible to conduct biomechanical *in vivo* experiments; additionally, *in vitro* experiments are not easy to carry out, because specimens are expensive and unavailable (Ames et al., 2005; Yuchi et al., 2019). Finite element analysis can deal with these limitations and obtain satisfactory results as well as detailed internal information of the cervical spine; furthermore, it can possibly provide interpretations for postoperative symptoms (Yuchi et al., 2019). As a digital research tool, finite element analysis can assess cervical spine kinematics or biomechanics, can simulate various clinical situations, and has been widely applied to spine biomechanical research so far (Wu W. et al., 2021; Sang et al., 2021).

In our study, we analyze the biomechanical characteristics after performing different minimally invasive surgeries in light of range of motion of segments and the pressure on the following structures: intervertebral disc, FJs, and UJs. The objective of this study is to assess and compare the cervical biomechanical performance of the four surgical models (PECF, MACF, ATd-ECD, and ATc-ECD) against the preoperative three-dimensional finite element model of normal cervical vertebrae C3–C7. After analyzing and comparing the changes of ROM, AFP, IDP, FJs, and UJs CPRESS between preoperation and postoperation, we expect to provide useful information for spine surgeons regarding the selection of operation plans.

## Materials and Methods

### Establishment of Three-Dimensional Model

**FIGURE 1:**
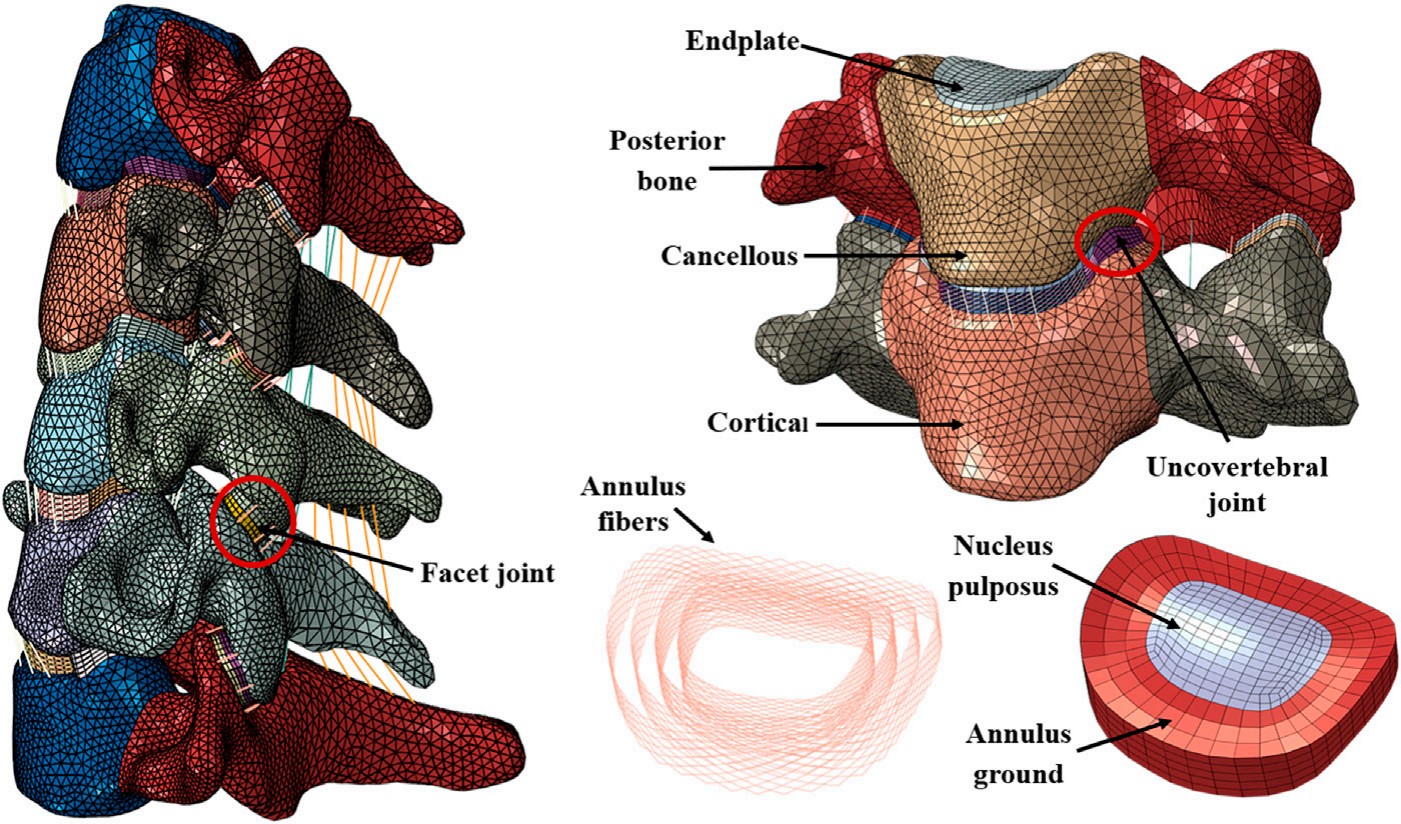
Finite element model of intact C3–C7 and components.

The data of the C3–C7 finite element model establishment stemmed from a computed tomography (CT) scan of a healthy subject (gender: male; age: 25; height: 176 cm; weight: 65 kg). The subject does not have any symptoms of cervical spondylopathy and feelings of neck discomfort. The conversion of CT data to STL files was conducted in Mimics 21.0 (Materialise Inc., Leuven, Belgium). Then, the STL files were imported into the Geomagic Wrap 2017 software (Geomagic, Inc., Research Triangle Park, NC, United States) for smoothing the original model. According to the anatomical position and shape of disc, UJs, and FJs, we used the SOLIDWORKS 2018 (Dassault Systèmes Inc., France) software to generate them step by step. The construction of the mesh model and the finite element preprocessing were done using HyperMesh 14.0 (Altair Engineering, Inc., Executive Park, CA, United States). Finally, the finite element model calculation and analysis were implemented with the Abaqus 2020 software (Abaqus, Inc., Providence, RI, United States). The intact model and its components are presented in Figure 1.

The intact finite element model consisted of the cortical bone, cancellous bone, posterior bone, endplate, annulus ground, annulus fibers, nucleus pulposus, FJ, UJ, and five major ligaments. The endplate was located between the intervertebral disc and cancellous bone. The cortical bone and endplate were assumed as 0.5 mm thin bony shells. The nucleus pulposus was defined as a non-compressible material, and the volume rate between nucleus pulposus and annulus ground was 4:6 (Wu et al., 2019; Hua et al., 2020; Sang et al., 2021). The annulus fibers accounted for approximately 20% of ground volume, and they were circumferential around the surface of the annulus ground at an angle of approximately 15°–45° with respect to the endplate horizontal plane (Mo et al., 2015). Based on the mesh model, five ligaments, i.e., anterior longitudinal ligament, posterior longitudinal ligament, capsular ligament, interspinous ligament, and ligamentum flavum, were constructed node to node.

Facet cartilages were split into two equal inferior and superior parts. The contact method between facet cartilages was modeled as frictional contact, with the surface-to-surface method. Additionally, the friction coefficient was 0.1 (Wong et al., 2020; Wo et al., 2021). The contact between UJs and cortical bone was identical to facet cartilages. For convenience reasons, we assumed the UJs as hexahedrons and neglected its anatomical fissure.

### Material Properties and Element Types

The intact finite element model comprised 254,406 elements and 67,194 nodes. Fibers and ligaments were simulated by tension-only truss elements. The vertebral body was simulated by four-node tetrahedral elements, and the rest of the material was simulated by eight-node hexahedral elements. The detailed material properties (Lee et al., 2011; Cai et al., 2020a; Guan et al., 2020; Hua et al., 2020; Ke et al., 2020; Wang et al., 2020; Sang et al., 2021; Wo et al., 2021) of all parts are presented in Table 1.

**TABLE 1:** The mechanical property of the components of the C3–C7 finite element model.

Components	Young’s modulus (MPa)	Poisson’s ratio	Element type	Cross-sectional area (mm^2^)
Cortical bone	12,000	0.3	C3D4	-
Cancellous bone	450	0.29	C3D4	-
Posterior bone	3,500	0.29	C3D4	-
Endplate	500	0.4	C3D8R	-
Annulus ground	3.4	0.4	C3D8H	-
Annulus fibers	110	0.3	Tension-only truss	-
Nucleus pulposus	1	0.49	C3D8H	-
Facet joints	10	0.4	C3D8R	-
Uncovertebral joints	10	0.4	C3D8R	-
Anterior longitudinal ligament	10	0.3	Tension-only truss	6
Posterior longitudinal ligament	10	0.3	Tension-only truss	5
Capsular ligament	10	0.3	Tension-only truss	46.6
Ligament flavum	1.5	0.3	Tension-only truss	5
Interspinous ligament	1.5	0.3	Tension-only truss	10

### Construction of Postoperative Models

**FIGURE 2:**
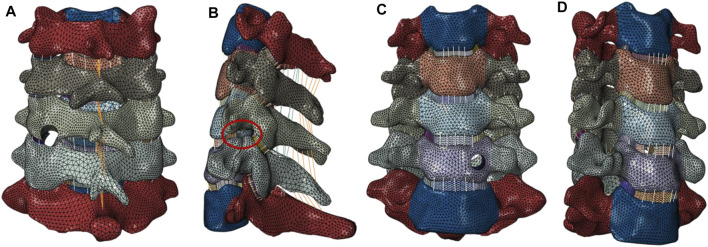
(**A**) PECF: a 7 mm diameter tunnel was constructed around the V-point at the C5–C6 left FJ; and partial articular capsular and laminae were removed. (**B**) MACF: the posterior part of the left UJ was removed at the C5–C6 segment without disc resection. (**C**) ATc-ECD: a 6 mm diameter tunnel was designed on the C6 vertebral body left side from anterior inferior towards posterior superior, and endoscopic instruments can reach the region of the compressed nerve root. (**D**) ATd-ECD: a 3.9 mm diameter tunnel was constructed at the C5–C6 intervertebral space from the right front to the left rear, and the target area was the left lateral recess.

All postoperative models were altered on the basis of the intact model. The four postoperative models are shown in Figure 2. A tunnel with a 3.9 mm diameter was established on the C5–C6 intervertebral disc from the right front to the left rear based on the intact model, and partial annulus fibers were removed in the building process of the ATd-ECD model. At the V-point of the C5–C6 left FJ, we built a 7 mm diameter tunnel from inside to outside, and partial capsular ligament and cartilage were removed to simulate posterior cervical foraminotomy in the PECF model. For the MACF model, we removed only the posterior part of UJs at C5–C6, without disc resection. For the ATc-ECD model, a 6 mm diameter tunnel was established from anterior–inferior towards posterior–superior of the C6 vertebral body; additionally, we did not interfere with the anterior longitudinal ligament and posterior longitudinal ligament during the process.

### Boundary and Loading Conditions

A 1 Nm moment was applied at the surface of the C3 superior endplate to imitate the movement of cervical spine flexion, extension, axial rotation, and lateral bending under a 50 N follower preload. The intermediate node of the endplate was coupled with each endplate surface, and these coupled nodes were connected by connector units (Cai et al., 2020b). The follower load was applied to each connector unit. The direction of the follower load was approximately tangential to the cervical spine physiological curve, and the follower load could provide a partial effect of muscle to the cervical movement (Bell et al., 2018). Hence, it could simulate physiological conditions better than the axial compressive load. We calculated the displacement of the intact model under moment and load during different motions; then, the displacement load was applied to every surgical model. The surface of the C7 inferior endplate is always immobilized completely when the cervical spine is in motion.

## Results

### Model Validation

**FIGURE 3:**
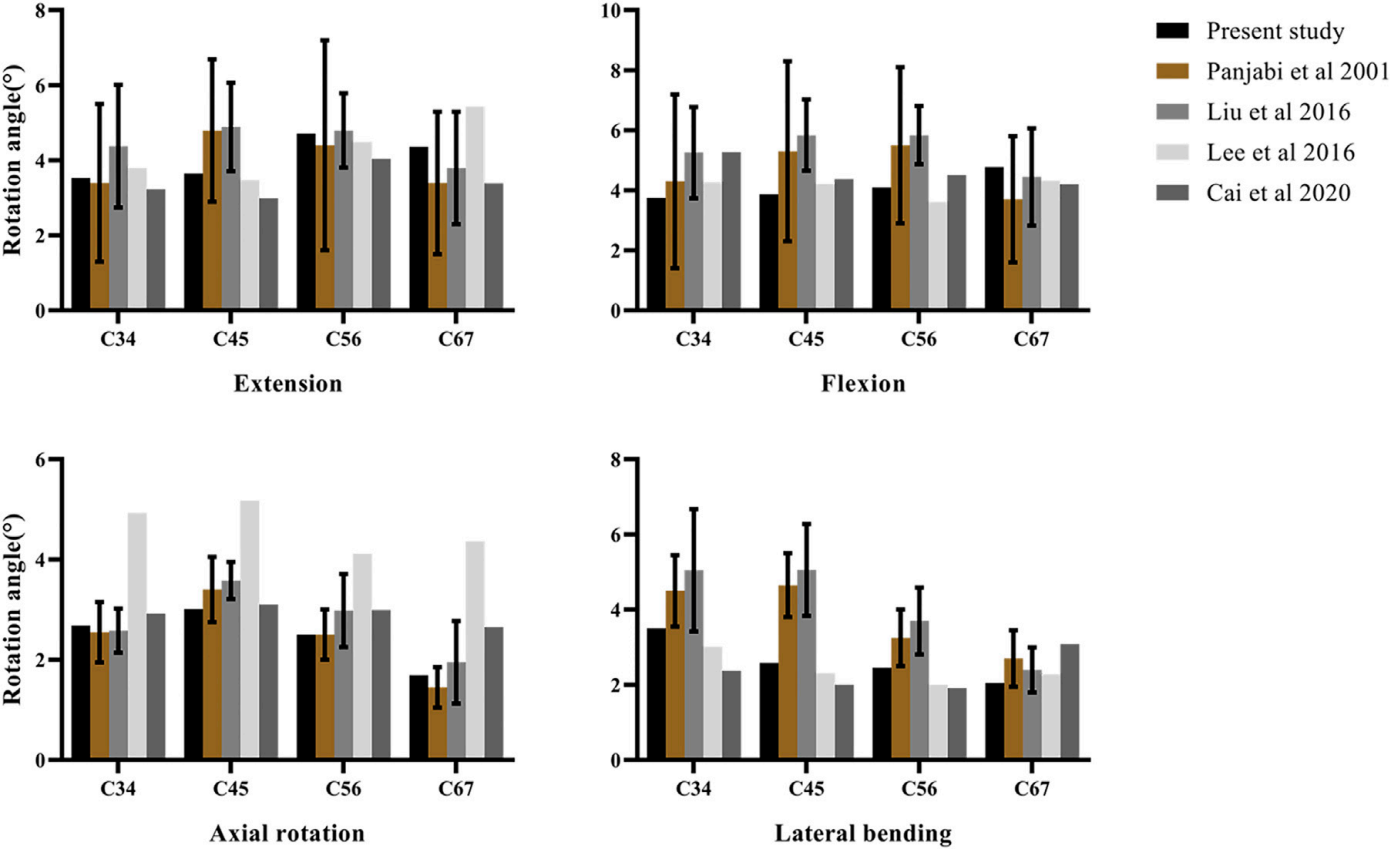
Validation of the intact C3–C7 finite element model.

The loading conditions of model validation were identical with the previous experiments. The ROM of segments of the C3–C7 finite element model was calculated under the condition of 1 Nm pure moment and compared with published data. The predicted ROM was consistent with *in vitro* experiments and previous finite element results (Panjabi et al., 2001; Lee et al., 2016; Liu et al., 2016; Cai et al., 2020a). The comparison between our results and previous ones is shown in Figure 3.

### ROM

**FIGURE 4:**
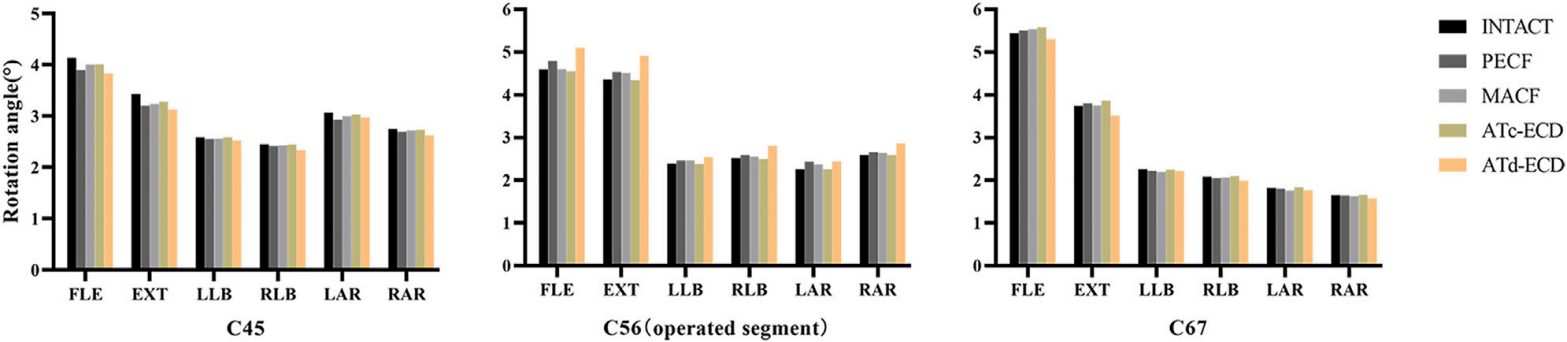
The ROM of C4–C5, C5–C6, and C6–C7 in the intact model and surgical models. FLE, flexion; EXT, extension; LLB, left lateral bending; RLB, right lateral bending; LAR, left axial rotation; RAR, right axial rotation.

The ROM of operated and adjacent segments is shown in Figure 4. The most significant increase of the operated segment ROM occurred in the ATd-ECD, and the ROM increased by 11.07%, 12.77%, 11.56%, and 10.51% during flexion, extension, right lateral bending, and right axial rotation, respectively. For the PECF model, we found that the ROM of the operated segment increased obviously during extension–flexion and axial rotation by 8.46% and 10.74%, respectively. For the MACF model, axial rotation and lateral bending generated more mobility in the operated segment, followed by extension motion, and the percentage change was 6.93%, 4.57%, and 3.56%, respectively. However, the ROM of the operated segment in ATc-ECD showed a tiny decrease. For adjacent segments, the ROM of four surgical models showed a decreasing trend compared with the intact model.

### AFP and IDP

**FIGURE 5:**
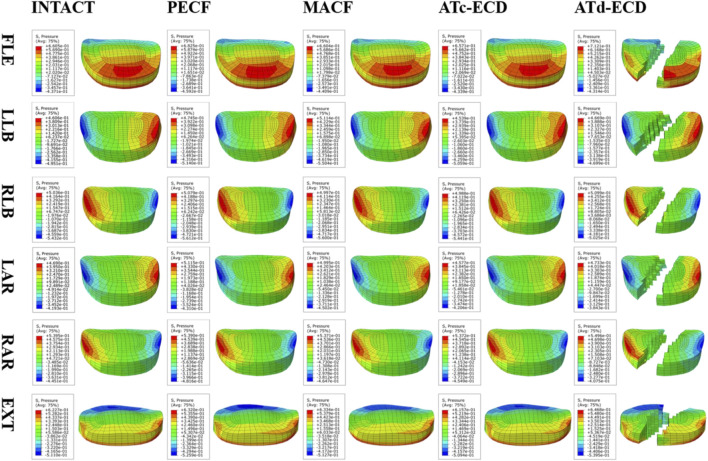
The intervertebral disc pressure contour plots of the operated segment during different motions. FLE, flexion; LLB, left lateral bending; RLB, right lateral bending; LAR, left axial rotation; RAR, right axial rotation; EXT, extension. The bottom of the figure describes the distribution of pressure of the posterior portion of the intervertebral disc in the contour plot of flexion. In contrast, in the contour plot of the rest of motions, the bottom of the figure describes the distribution of pressure of the anterior portion of the intervertebral disc in the contour plot of flexion.

**FIGURE 6:**
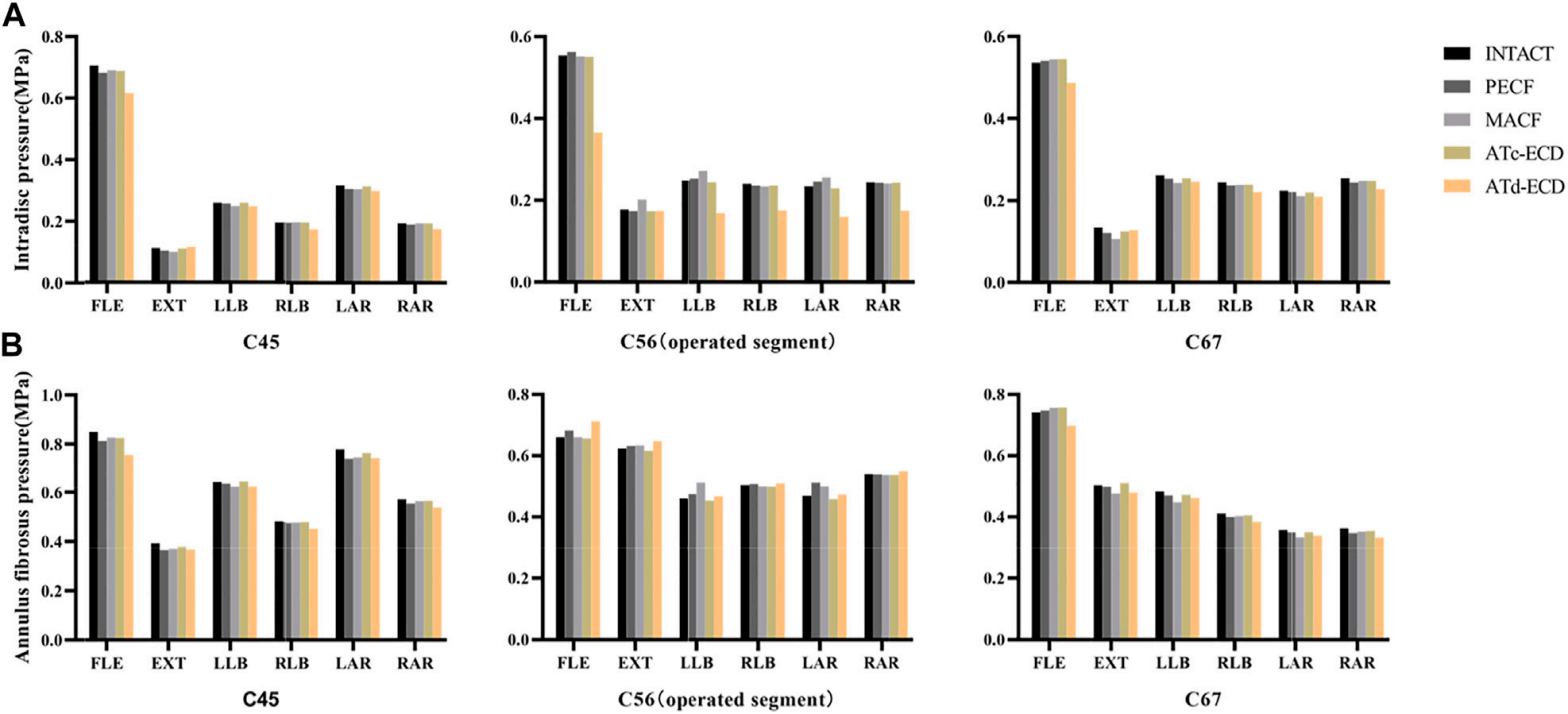
The AFP and IDP of C4–C5, C5–C6, and C6–C7 in the intact model and surgical models. (**A**) Intradiscal pressure. (**B**) Annulus fibrosus pressure. FLE, flexion; EXT, extension; LLB, left lateral bending; RLB, right lateral bending; LAR, left axial rotation; RAR, right axial rotation.

The contour plots of the intervertebral disc pressure at C5–C6 are shown in Figure 5, and the IDP and AFP variations of different models are shown in Figure 6. In comparison with the other models, the IDP of the operated segment significantly increased in the MACF model, and the IDP increased by 13.95%, 9.75%, and 9.19% during extension, left lateral bending, and left axial rotation, respectively. For the PECF model, the IDP of the operated segment increased obviously during left axial rotation, and the increase rate was 4.96%. On the contrary, in the ATc-ECD and ATd-ECD models, the IDP of the operated segment decreased during different motions. The IDP of the operated segment decreased by 34.01%, 32.07%, 27.19%, 31.51%, and 28.32% during flexion, left lateral bending, right lateral bending, left axial rotation, and right axial rotation, respectively, in the ATd-ECD model; on the other hand, in the ATc-ECD model, the maximum percentage of decrease was only 2.09% during extension. The variation trend of the AFP was similar with the IDP. Different from the IDP variation, in the ATd-ECD model, the AFP of the operated segment increased in different motions, and the maximum increase rate was 7.81% during flexion motion. For the PECF model, the AFP of the operated segment increased significantly during left axial rotation by 9.06%. For the MACF model, the AFP of the operated segment increased significantly during left lateral bending by 11.03%. However, the AFP and the IDP decreased in the adjacent segments.

### FJs and UJs CPRESS

**FIGURE 7:**
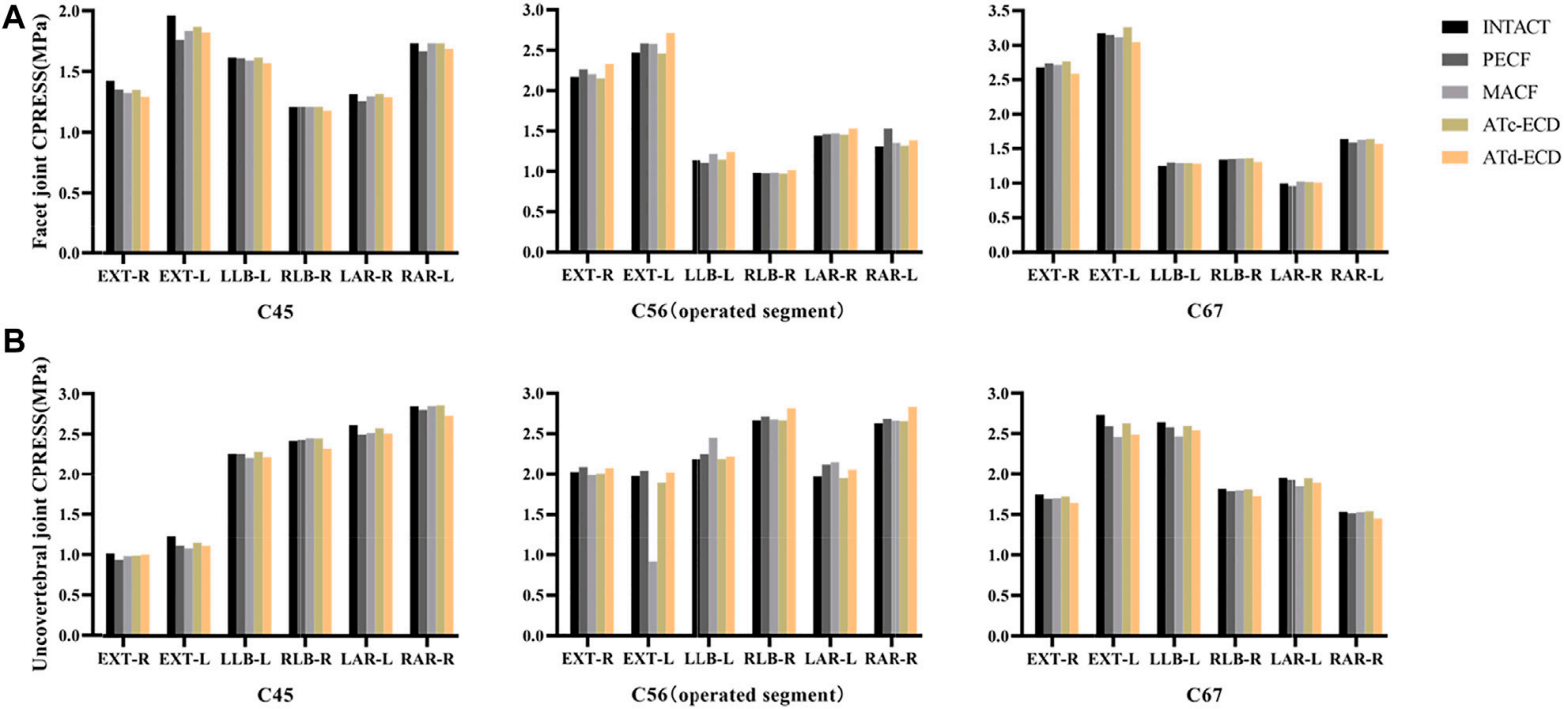
The FJs CPRESS and UJs CPRESS of C4–C5, C5–C6, and C6–C7 in the intact model and surgical models. (**A**) -R, right facet joint; -L, left facet joint. (**B**) -R, right uncovertebral joint; -L, left uncovertebral joint. Ipsilateral UJ and FJ bear major pressure during lateral bending, whereas ipsilateral UJ and contralateral FJ bear major pressure during axial rotation. FLE, flexion; EXT, extension; LLB, left lateral bending; RLB, right lateral bending; LAR, left axial rotation; RAR, right axial rotation.

The FJs and UJs CPRESS variations of different models are shown in Figure 7. According to the results, the increase of the operated segment FJs CPRESS in the ATd-ECD was apparent compared with the other models under different motions. For the ATc-ECD model, the variation of FJs CPRESS was inconspicuous. For all models, the PECF generated the maximum operated segment FJs CPRESS during right axial rotation, with a percentage increase of 16.77%. The results suggest that different surgical models had lower effect on UJs. During lateral bending and axial rotation, the ipsilateral UJs CPRESS of the operated segment increased significantly when the cervical spine moved towards the surgical side in the MACF model, and the percentages were 12.24% and 8.82%, respectively, whereas the left UJs CPRESS decreased by 53.47% during extension. For the PECF model, the UJs CPRESS of the operated segment increased by 9.48% during rotation. The changes of FJs and UJs CPRESS in adjacent segments were similar with the ones in ROM and IDP.

## Discussion

This study uses an indirectly validated three-dimensional finite element model of a normal C3–C7 segment to simulate cervical minimally invasive surgeries, including PECF, MACF, ATc-ECD, and ATd-ECD. Through comparison and analysis of the biomechanical changes of the four minimally invasive surgeries models, we hope to provide some evidences for surgeons to support their selection of appropriate surgical plans. From previous research (Ren et al., 2019; Yuchi et al., 2019; Chen et al., 2020; Ke et al., 2020), the assessment of biomechanics includes the following parameters: ROM, IDP, AFP, and FJs CPRESS. However, the biomechanical characteristics of UJs, which are a unique structure of the cervical spine, are not completely clear after minimally invasive surgeries. Therefore, in our study, we constructed the structure of UJs in the finite element model and calculated the contact pressure of UJs in the postoperative condition.

With regard to the ROM, the ATc-ECD model was closest to the intact model. Umebayashi et al. (2013) calculated the ROM of the functional spinal unit in the ATc-ECD using radiography measurements; the values were 5.0 ± 2.7° and 4.2 ± 3.9° in preoperative and postoperative conditions, respectively, and there was no significant difference for the ROM of the functional spinal unit between the two conditions. A similar situation occurred in our ATc-ECD model, whereas the ATd-ECD model significantly increased the segmental ROM compared with the other models, which was in accordance with existing studies (Yuchi et al., 2019; Chen et al., 2020). For the PECF model, part of the left-side FJ was removed at the C5–C6 segment, and FJs play an important role in limiting rotation and extension–flexion motion (Cai et al., 2020b). Similarly, the PECF significantly increased the operated segment ROM during rotation and extension–flexion and was lacking stability compared to the MACF model in our study. For the MACF model, the posterior part of the left UJ was resected. UJs are regarded as a stabilizer to limit cervical posterior translation and lateral bending; however, the function is undefined in the lower cervical spine, and there is a hypothesis that UJs provide more stability in rotation (Penning and Wilmink, 1987). Clausen et al. (1997) reported that the ROM on the C5–C6 segment increased by 25% and 14% during torsion and lateral bending, respectively, after UJ resection. Our study also supported this hypothesis by removing the posterior part of the left UJs in the MACF model. This is probably because of the greater disc facet angle in the lower cervical spine and UJs may permit more cervical torsion movement (Milne, 1991). Due to the surgical models and the intact model moving with the same displacement, the ROM of adjacent segments performed a compensatory decrease, which may be related to the increase of the operated segment (Chen et al., 2020). Hence, judging from the biomechanical viewpoint, the risk of adjacent segment degeneration might greatly decrease compared with anterior cervical discectomy and fusion after minimally invasive surgeries.

With regard to IDP and AFP, the MACF significantly increased, followed by the PECF model. We speculated that the UJs, which are load-bearing structures, might have a close relationship with the intervertebral disc. After removal of the UJs, there is a shift of a more compressive load from UJs to the neighboring intervertebral disc. Besides, an equilibrium mechanism may exit in the MACF and PECF. Rotation or lateral bending towards the direction of the surgical side caused IDP and AFP to increase, and the IDP and AFP decreased when the cervical spine shifted towards the opposite direction. Our results suggest that the ATc-ECD caused a very small intradiscal pressure decrease. However, the IDP decreased significantly after conducting the ATd-ECD, which was consistent with the previous research of Sun and Chen (Chen et al., 2020; Sun et al., 2021), and the situation of the AFP increase was consistent with the study by Yuchi et al. (2019). With the decrease of IDP, the capacity of the nucleus pulposus of supporting the load decreased. Afterwards the compressive load is transferred to the annulus fibrosus, and the height of the intervertebral disc will not be maintained (Sun et al., 2021). For that reason, the height of the intervertebral disc decreased in previous clinical studies after performing the ATd-ECD.

For the FJs CPRESS, the model of the four minimally invasive surgeries showed an increase of a different magnitude in the operated segment, and the ATd-ECD model obtained maximum CPRESS. The increase of the CPRESS might cause joint abrasion and accelerate joint degeneration (Yuchi et al., 2019). When the PECF occurred in extension and rotation motion, the CPRESS of FJs increased significantly but decreased in lateral bending; this result is inconsistent with the study by Yuchi et al. (2019). This could be because of the ROM and contact area of FJs increasing during rotation and extension. Except for the ATc-ECD, the rest of the models may cause UJ degeneration at the operated segment, especially in rotation and lateral bending motions. However, the UJs CPRESS of the surgical side in the MACF decreased considerably during extension. We consider that the posterior part of UJs contributes importantly to stability (Kotani et al., 1998) and that it is the concentration area of pressure during extension. Therefore, the contact pressure showed an obvious decrease when removing the posterior part of UJs.

Furthermore, the ATd-ECD may result in irreversible iatrogenic disc injury and poorer cervical stability; still, Sun et al. (2021) reported that cervical stability was affected by the approach of angle and surgical diameter when conducting the ATd-ECD. In their study, cervical stability changed minimally when the angle of approach was 90°. For building a targeted channel and reducing disc injury as much as possible, surgeons often need a contrast agent mixed with methylenum coeruleum; nevertheless, the stain or contrast agent may bring some potential risk, such as toxic effect and discitis (Yang et al., 2014). Meanwhile, the intervertebral disc height cannot be lower than the endoscope diameter, which is currently 3–4 mm; thus, an excessively low disc height is a restriction (Ahn, 2016; Ren et al., 2020). The ATc-ECD and ATd-ECD are similar in technology. Previous studies have demonstrated that the ATc-ECD has hardly any impact on vertebrae strength if surgeons choose an endoscopic system with a diameter within 6 mm and a lateral approach (Umebayashi et al., 2013; Wu et al., 2018). Also, Ren et al. (2020) reported that bone defect within 6 mm could heal after the ATc-ECD, and the complete healing rate reached up to 94.28%. Therefore, in this study, we focus more on segmental stability rather than bone strength.

Our results show that the MACF can maintain segmental stability well, but there are some setbacks to its use. Considering that the vertebral artery is closely located at the UJ lateral side, vertebral artery injury could be a serious intraoperative complication when performing the MACF (Saringer et al., 2002). In addition, the distance between the lateral wall of the uncinate process and artery is disparate at different segments. Kim et al. (2012) reported that the shortest distance is located at the C3–C4 segment, and the farthest distance is located at the C6–C7 segment. This means that the MACF procedure cannot be carried out at each segment, and surgeons need to prudentially estimate the positional relationship between UJs and vertebral artery before operation. Meanwhile, it is noteworthy that the gap between UJs and vertebral artery displays a constriction when UJ hypertrophy or serious disc degeneration occurs (Urbanschitz et al., 2021). Hence, the risk of vertebral artery injury may further increase.

The spine is formed from complex and interrelated structures containing the vertebral body, FJ, intervertebral disc, ligaments, and muscle tissue, which together contribute to spine stability and transmission of force, and one structure degeneration or injury will affect the rest of the structures (Kumaresan et al., 2001; O'Leary et al., 2018; Vergroesen et al., 2015). In the present study, the standard model was constructed on the basis of a healthy cervical spine rather than a degenerated cervical spine. A degenerated spine has a decreased segmental motion and intradiscal pressure and increased FJ load compared with a healthy spine (Hussain et al., 2010; Vergroesen et al., 2015; Cai et al., 2020a). Hence, there are differences between healthy and pathological postoperation models in the calculated parameters, including ROM, CPRESS, and intervertebral disc pressure. And it will cause some deviations such that finite element analysis results completely substitute for a variety of pathologic postoperative outcomes. However, the morphology of a pathological cervical spine is diverse, including different cervical curvatures and intervertebral disc height loss or not, which causes the diversification of biomechanical results after operation. In addition, the indications of different minimally invasive surgeries are not all the same, which means there are restrictions in choosing an appropriate pathological model. Though lacking individual specificity, CT data of a healthy male contribute to the unification of variables, and the study results are still universal and adaptable. Our results might be closer to some clinical cases that suffer from pure cervical disc herniation without apparent structure change of the cervical spine.

There are several limitations in our study that should be reported. Firstly, the validation method of the standard model is an indirect way of comparison with published data. Although most finite element analyses of the spine used indirect validation (Jones and Wilcox, 2008), the accuracy and precision of finite element results decreased because of unclear *in vitro* experiment conditions, large standard deviation, and lack of specific material properties. Thus, the calculated pressures of the intervertebral disc and articular cartilage are not equal to the actual value. Combined with identical data of *in vitro* experiments, developing a specific finite element model might be a trend. Secondly, although the follow load could provide a part effect of muscle to the cervical spine, the detailed and complicated function of muscle-to-spine movement cannot be simulated. Thirdly, there is a lack of recognized method of constructing UJs. We simplified UJs in the model, which might lead to loss of some details. Fourthly, for good convergence in the calculating process, we chose homogeneous and linear materials. Although they have partial impact on the localized mechanical environment, they do not affect the cervical spine kinetics. The material properties need to be noticed if the direction of the study changes. Overall, our predicted results might not represent the precise clinical numerical value, but they could predict a dependable variation tendency during postoperative cervical surgery in different motions. For obtaining comprehensive and accurate biomechanical data, more *in vivo* and *in vitro* experiments should be conducted in the future.

## Conclusion

In our study, all types of minimally invasive surgeries displayed good biomechanical stability. From the standpoint of biomechanics, the physiological status of the ATc-ECD was close to the normal cervical model, followed by the MACF model. The ATd-ECD model might significantly accelerate disc and joint degeneration compared with the other three types of minimally invasive surgeries. Considering safety and conditions of application, the PECF and ATc-ECD may be relatively suitable techniques for cervical spondylotic radiculopathy. Certainly, surgeons can choose the appropriate procedures according to their proficiency level and radiological results, such as intervertebral disc height and types of disc herniation.

## Data Availability

The original contributions presented in the study are included in the article/Supplementary Material, further inquiries can be directed to the corresponding author.

## Abbreviations

AFP, annulus fibrosus pressure; ATd-ECD, anterior transdiscal approach of endoscopic cervical discectomy; ATc-ECD, anterior transcorporeal approach of endoscopic cervical discectomy; CPRESS, contact pressure; CT, computed tomography; EXT, extension; FLE, flexion; FJs, facet joints; IDP, intradiscal pressure; LAR, left axial rotation; LLB, left lateral bending; MACF, microsurgical anterior cervical foraminotomy; PECF, posterior endoscopic cervical foraminotomy; RAR, right axial rotation; RLB, right lateral bending; ROM, rang of motion; UJs, uncovertebral joints.
