# Corrigendum: Comparative Analysis of the Biomechanics Characteristics After Different Minimally Invasive Surgeries for Cervical Spondylopathy: A Finite Element Analysis

**DOI:** 10.3389/fbioe.2022.843486

**Published:** 2022-02-16

**Authors:** Tao He, Jun Zhang, Tong Yu, Jiuping Wu, Tianyang Yuan, Rui Liu, Zhihe Yun, Haorui Du, Le Qi, Junyan An, Wu Xue, Xinyu Nie, Qinyi Liu

**Affiliations:** Department of Spine Surgery, The Second Hospital of Jilin University, Jilin University, Changchun, China

**Keywords:** anterior transdiscal approach of endoscopic cervical discectomy, posterior endoscopic cervical foraminotomy, microsurgical anterior cervical foraminotomy, anterior transcorporeal approach of endoscopic cervical discectomy, cervical minimally invasive surgery, biomechanics, finite element analysis

In the original article, there was a mistake in Figures 1, 6, 7 as published. In Figure 1, the tagging of the “annulus ground” and “nucleus pulposus” labels was mistakenly reversed. In Figure 6, the most lateral column in the histogram of C67 AFP (for Annulus fibrosus pressure in Figure 6B), is redundant and this column should be deleted. In Figure 7, in the histogram of C67 Facet joint CPRESS in Figure 7A, the columns of ATc-ECD and ATd-ECD were mistakenly reversed. The corrected Figures 1, 6, 7 appear below.

**FIGURE 1:**
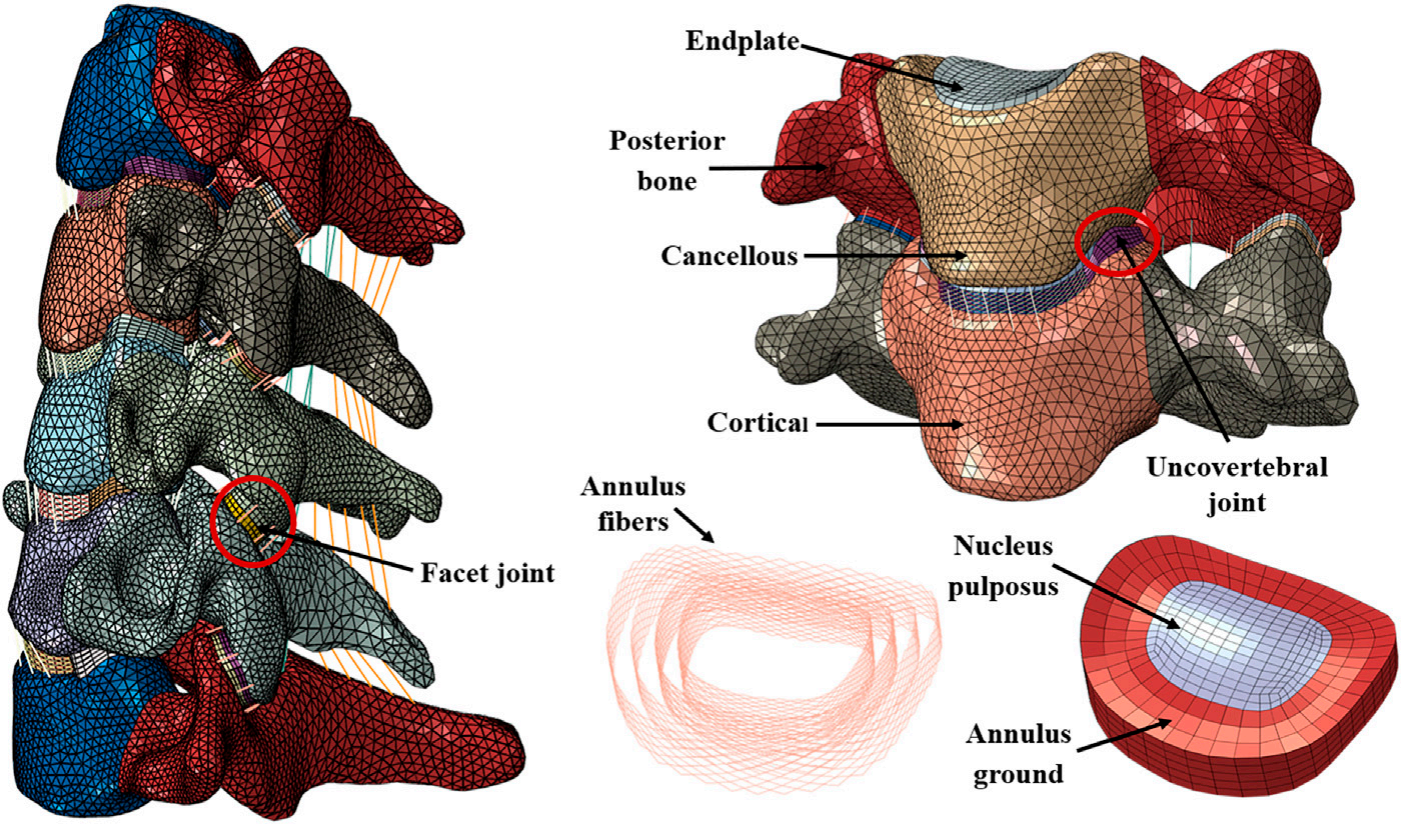
Finite element model of intact C3–C7 and components.

**FIGURE 6:**
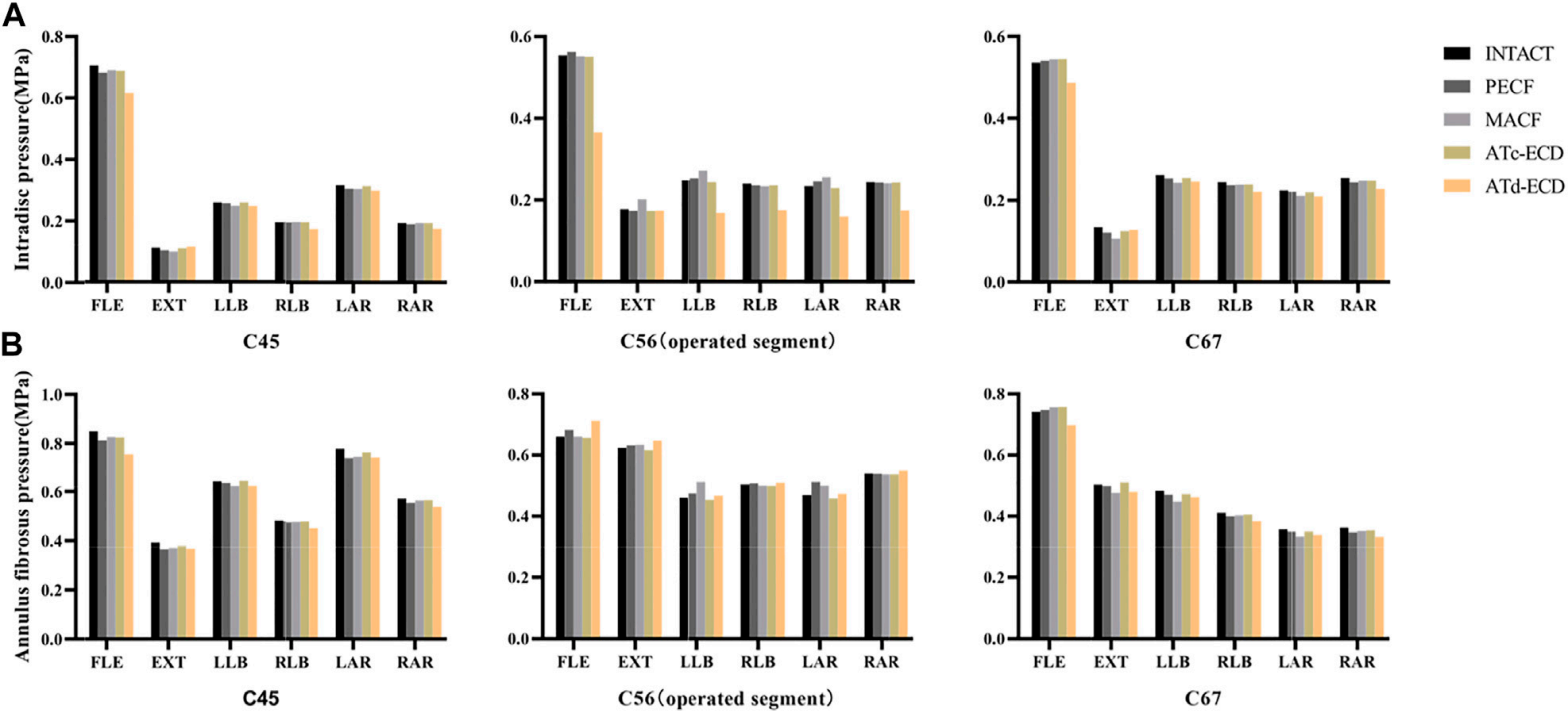
The AFP and IDP of C4–C5, C5–C6, C6–C7 in intact model and surgical models. **(A)** Intradiscal pressure; **(B)** Annulus fibrosus pressure. FLE, flexion; EXT, extension; LLB, left lateral bending; RLB, right lateral bending; LAR, left axial rotation; RAR, right axial rotation.

**FIGURE 7:**
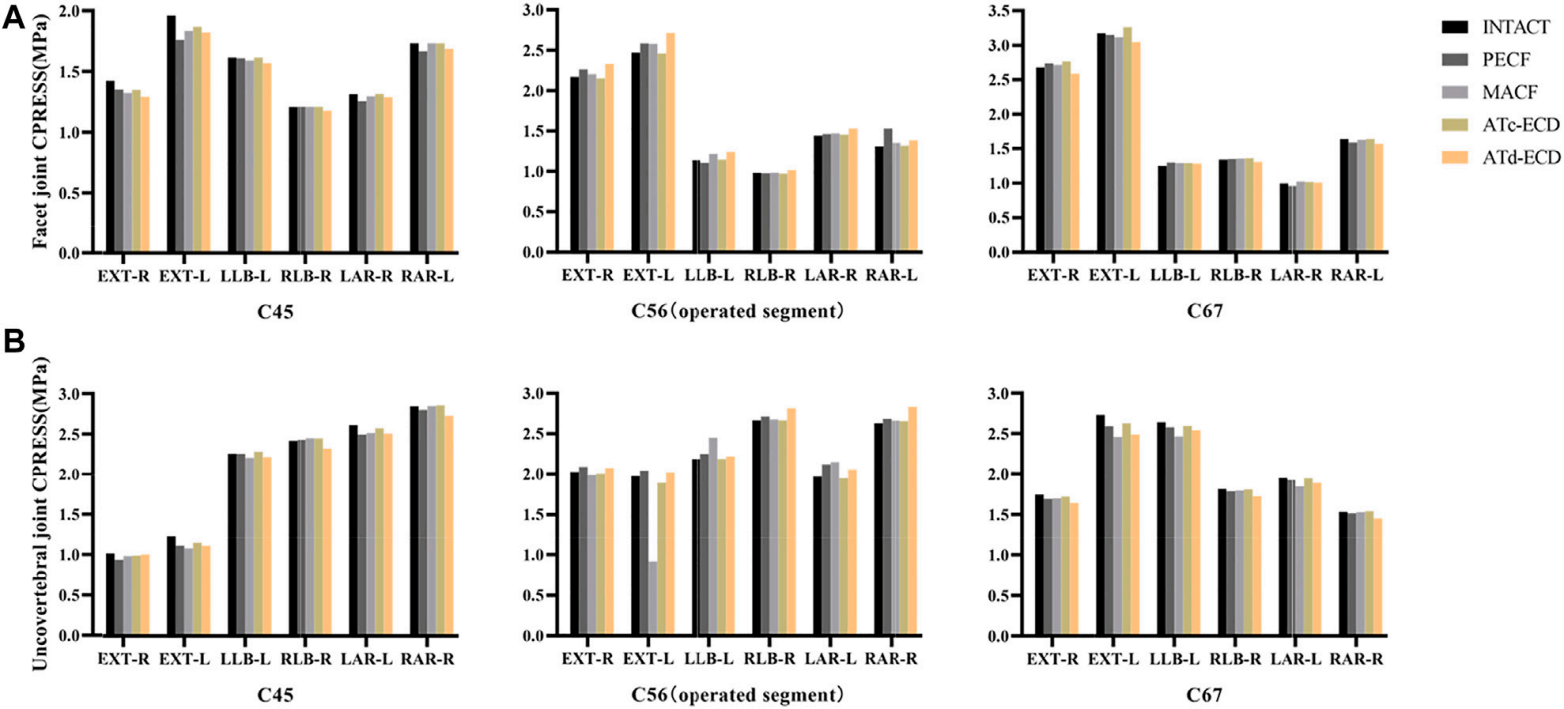
The FJs CPRESS and UJs CPRESS of C4–C5, C5–C6, C6–C7 in intact model and surgical models. **(A)** -R, right facet joint; -L, left facet joint. **(B)** -R, right uncovertebral joint; -L, left uncovertebral joint. Ipsilateral uncovertebral joint and facet joint bear major pressure during lateral bending, whereas Ipsilateral uncovertebral joint and contralateral facet joint bear major pressure during axial rotation. FLE, flexion; EXT, extension; LLB, left lateral bending; RLB, right lateral bending; LAR, left axial rotation; RAR, right axial rotation.

Additionally, in the **Results** section, subsection **Model Validation**, the citation **Lee et al., 2011** is incorrect and should be written as Lee et al., 2016. The full reference details for Lee et al., 2016 have been included below.

In the **Results** section, subsection **AFP and IDP**, paragraph 1, the term “lateral axial rotation” has been corrected to “left axial rotation.” Lastly, in the **Results** section, subsection **FJs and UJs CPRESS**, paragraph 1, UJs CPRESS decreased by 53.47% during extension and not during flexion, as originally published. These have been corrected in the main text.

The authors apologize for these errors and state that they does not change the scientific conclusions of the article in any way. The original article has been updated.
